# Predictors of Technical Success and Rate of Complications of Image-Guided Percutaneous Transthoracic Lung Needle Biopsy of Pulmonary Tumors

**DOI:** 10.1371/journal.pone.0124947

**Published:** 2015-04-09

**Authors:** Stephan Otto, Birger Mensel, Nele Friedrich, Sophia Schäfer, Christoph Mahlke, Wolfram von Bernstorff, Karen Bock, Norbert Hosten, Jens-Peter Kühn

**Affiliations:** 1 Department of Diagnostic Radiology and Neuroradiology, University Medicine Greifswald, Greifswald, Germany; 2 Institute of Clinical Chemistry and Laboratory Medicine, University Medicine Greifswald, Greifswald, Germany; 3 Department of Surgery, Division of General, Visceral, Thoracic and Vascular Surgery, University Medicine Greifswald, Greifswald, Germany; University of Utah Health Sciences Center, UNITED STATES

## Abstract

**Purpose:**

To investigate predictors of technical success and complications of computed tomography (CT)-guided percutaneous transthoracic needle biopsy of potentially malignant pulmonary tumors.

**Material and Methods:**

From 2008 to 2009, technical success and rate of complications of CT-guided percutaneous transthoracic lung needle biopsies of patients with suspicious pulmonary tumors were retrospectively evaluated. The influence on technical success and rate of complications was assessed for intervention-related predictors (lesion diameter, length of biopsy pathway, number of pleural transgressions, and needle size) and patient-related predictors (age, gender, reduced lung function). In addition, technical success and rate of complications were compared between different interventional radiologists.

**Results:**

One hundred thirty-eight patients underwent biopsies by 15 interventional radiologists. The overall technical success rate was 84.1% and was significantly different between interventional radiologists (range 25%-100%; p<0.01). Intervention-related and patient-related predictors did not influence the technical success rate. The overall complication rate was 59.4% with 39.1% minor complications and 21.0% major complications. The rate of complications was influenced by lesion diameter and distance of biopsy pathway. Interventional radiologist-related rates of complications were not statistically different.

**Conclusions:**

Technical success of percutaneous, transthoracic lung needle biopsies of pulmonary tumors is probably dependent on the interventional radiologist. In addition, lesion diameter and length of biopsy pathway are predictors of the rate of complications.

## Introduction

The treatment of pulmonary tumors depends on their malignant potential. Core biopsies with histopathologic analyses are the gold standard for the characterization of pulmonary lesions. Image-guided percutaneous transthoracic lung needle biopsy of pulmonary lesions is a minimally invasive procedure. It is employed if other techniques like transbronchial biopsies are not feasible or have failed [1,2].

Computed tomography (CT)-guided percutaneous transthoracic lung needle biopsy is a clinically accepted technique for sampling lung tumors. The main purpose of this technique is to obtain a sufficiently large tissue sample without procedure-associated complications. To achieve this aim, it is important to know predictors potentially influencing the outcome of percutaneous transthoracic lung biopsies. Only few reports have been published on interventional-related as well as patient-related predictors of technical success and rate of complications of percutaneous biopsies of pulmonary nodules [3–6].

In addition, most published data were obtained during clinical studies. This included strict standardized protocols allowing only one or two interventional radiologists to perform the procedures. However, in our opinion, this does not reflect the daily situation in hospitals. Data on the interventional radiologist-related outcome such as technical success and rate of complications are scare. We believe that the experience and skill of the interventional radiologist may substantially compromise the technical success and rate of complications of percutaneous transthoracic lung biopsies.

Therefore, we have analyzed potential predictors as well as the impact of different observers on the outcome of percutaneous transthoracic lung needle biopsies of potential malignant lung lesions.

## Material and Methods

The human study was approved by the local ethics committee of the University of Greifswald (BB 007/15). All CT-guided percutaneous transthoracic lung biopsies from 2008 to 2009 were retrospectively assessed. Percutaneous transthoracic lung biopsies of potential malignant lesions were obtained for further histopathological characterization. Indications were established in an interdisciplinary tumor board. Feasibility of the biopsy procedure was confirmed by interventional radiologists having more than five years of experience in interventional radiology. Written informed consent for percutaneous transthoracic lung biopsy was obtained at least 24 hours before the procedures began.

### Interventional Procedure

Interventional radiologists performed all percutaneous biopsies. Before beginning the procedure the interventional strategy, especially patient’s positions and biopsy pathways were planned using CT images. The interventions were performed in two different CT scanners (GE Lightspeed 8 slice, GE-Healthcare; Somatom Sensation 16 slice, Siemens Healthcare).

The intervention was started with skin disinfection and subcutaneous local anesthesia using 20 ml lidocaine 1% (Xylocitin, Jenapharm, Germany). First, a needle for local anesthesia was advanced toward the planned pathway. Second, following a small incision, the suspicious tumor was biopsied using a semi-automated side-cutting biopsy needle (Quick-Core Biopsy needle, Cook Medical USA) with a throw length of 2 cm. The interventional radiologist was responsible for selecting the size of the biopsy needle ranging from 14–18 Gauge. Afterwards, the correct positioning of the biopsy needle was confirmed by a CT scan. It was at the discretion of the interventional radiologist to take more than one sample of the tumor.

After completing the procedure, patients were instructed to rest for 24 hours. Blood pressure was measured hourly for a period of four hours. An expiratory chest X-ray was performed four hours after biopsy to exclude intervention-related complications in particular a pneumothorax.

### Data Analysis

The endpoints of this study were defined as follows:

#### Technical Success

Technical success was defined as completion of the biopsy procedure with histopathologic confirmation of a malignant pulmonary lesion.

When histopathology yielded the diagnosis of a benign lesion, a 3–6 month follow-up was performed. In those cases without changes of the tumor size the technical success of these benign lesions was finally verified.

#### Rate of Complications

The rate of complications included “minor” and “major” complications. Complications were assessed during patients’ hospitalization. Most complications, such as pneumothorax and/or pulmonary bleeding, were assessed using the CT scan obtained directly after the intervention as well as the X-ray obtained four hours after the intervention. In addition, secondary infections were documented during the period of hospitalization.

- *Minor Complication*: Intervention-related complication not requiring treatment, e.g., small pneumothoraces resolving spontaneously.
- *Major Complication*: Intervention-related complication requiring treatment or prolonging hospital stay, e.g., pneumothorax requiring a chest tube.

In addition, possible predictors influencing technical success and rate of complications were defined as follows:

*Intervention-related*: lesion size (maximum lesion diameter), length of interventional pathway (maximum distance from of skin to the center of the lesion), needle size (14–18 Gauge), number of pleural transgressions (one, more than one).
*Patient-related*: age, gender, and structural lung disease, such as emphysema, defined by imaging [7].
*Interventional radiologist-related*: differences in outcome between interventional radiologists.

### Statistics

Continuous data are given as mean ± standard deviation. Technical success and rate of complications are given as percentages.

Distributions of continuous parameters are presented as grouped box plots. Significant differences between groups were identified using a Wilcoxon rank-sum test. In addition, binary and multinominal logistic regression models were used to calculate odds ratios with 95% confidence intervals for technical success as well as presence of complications and their potential predictors. Statistical analysis was performed using SAS, version 9.3 (SAS statistical software, version 9.3, SAS Institute, Inc; Cary; NC, USA).

## Results

A total of 138 patients with potentially malignant pulmonary tumors underwent a CT-guided biopsy. The study population included 98 men and 40 women. Patients had a mean age of 66.6 ± 10.7 years. Pulmonary emphysema was identified in 71 patients (51.5%).

The pulmonary tumors had a mean diameter of 4.1 ± 2.5 cm with a range of 1.0–11.5 cm. The planned pathway for biopsy from skin to lesion had a mean length of 9.0 ± 2.2 cm with a range of 3.5–15.0 cm. Following needle sizes were used: 14 Gauge in 17 patients (12.3%), 16 Gauge in 69 patients (50.0%), and 18 Gauge in 52 patients (37.7%). The number of pleural transgressions per biopsy was one in 68 patients, two in 57 patients, three in 10 patients, and four or more in 3 patients.

An overall technical success was achieved in 116 patients (84.1%) leading to a final histopathological diagnosis. Biopsy was nondiagnostic in 22 patients. In these cases, tumors were characterized by repeat biopsy (n = 12) or surgery (n = 8), or malignancy was excluded by follow-up (n = 2). Histopathology revealed 121 malignant pulmonary lesions and 17 benign lesions.

There were 91 complications in 83 patients (60.1%)- 62 minor complications (n = 54 patients; 39.1%) and 29 major complications (n = 29 patients; 21.0%). The complications were pulmonary bleeding in 21 cases (15.0%), pneumothorax in 68 cases (49.3%), and hemoptysis in 2 cases (1.5%). 43% (n = 29) of patients with a pneumothorax required a chest tube (21% of the entire series).

**Fig 1:**
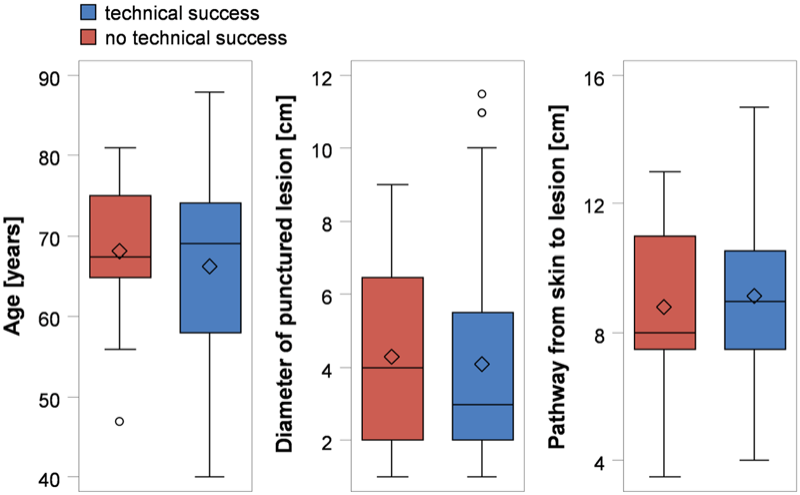
Technical success rates of CT-guided percutaneous transthoracic lung biopsies of pulmonary tumors according to patient age, lesion diameter, and length of interventional pathway.

**Fig 2:**
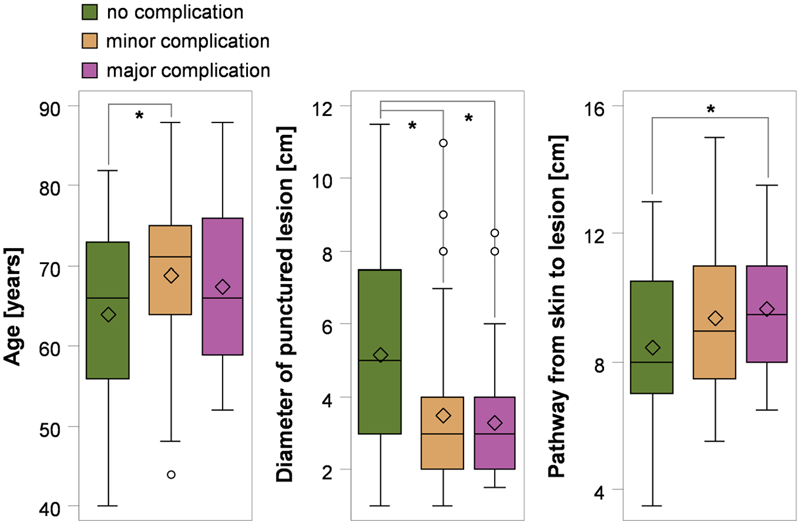
Predictors of complication rates of CT-guided percutaneous transthoracic lung biopsies of pulmonary tumors according to patient age, lesion diameter, and length of interventional pathway (* p<0.05).

**Fig 3:**
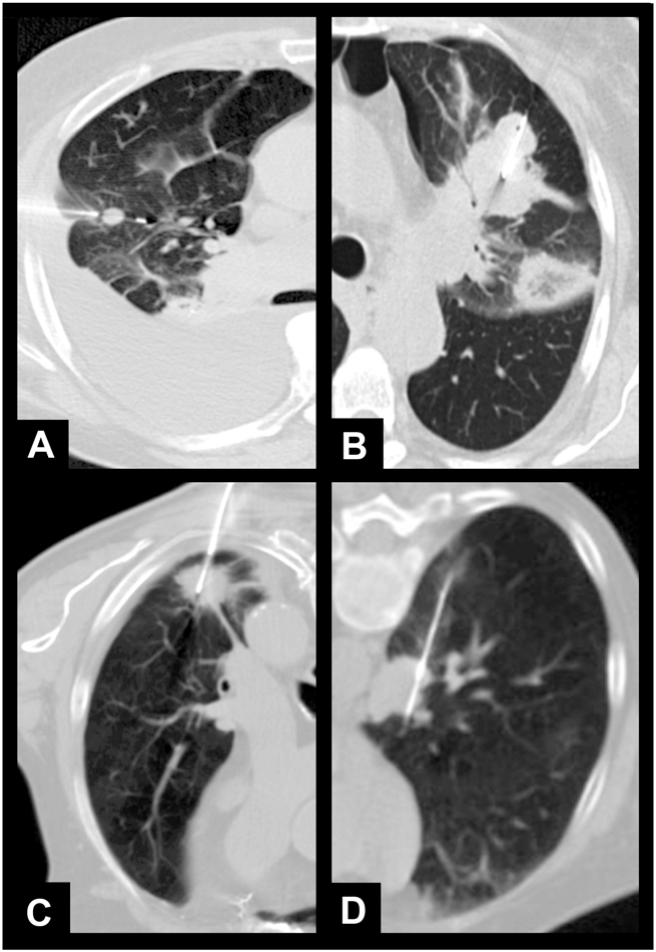
Examples of lung tumors of different sizes, sites, and lengths of interventional pathways. A) Small lesion; pathology: metastasis of colorectal cancer. B) Large lesion; pathology: lung carcinoma. C) Peripheral lesion; pathology: lung carcinoma. D) Central tumor; pathology: B-cell lymphoma.

Patient’s age, lesion diameter, length of biopsy pathway, number of biopsies per session, and presence of emphysema had no influence on technical success (Table 1, Fig 1). However, age, lesion diameter, and length of biopsy pathway had a significant effect on the occurrence of complications (Table 1, Fig 2). Patients with minor complications were on median 4 years older (Fig 2). The odds ratio of complications (minor & major) generally increased by 4% per year (Table 1). Furthermore, a longer pathway from the skin to the lesion was related to a 24% higher odds ratio of complications. Lesion diameter was 2 cm smaller in patients with complications, resulting in a 25% reduced odds ratio of complications per cm increase in lesion diameter. Different kinds of tumors and their biopsy procedures are shown in Fig 3.

**Table 1:** Predictors of percutaneous transthoracic lung biopsies of potentially malignant pulmonary tumors: odds ratios (OR) with 95% confidence interval (CI) for technical success and complications (minor and major complications).

	Technical success*		Complications*		Minor complications**		Major complications**	
	OR (95%-CI)	p	OR (95%-CI)	p	*OR (95%-CI)*	*p*	*OR (95%-CI)*	*p*
Age, per year	0.98 (0.94; 1.03)	0.45	**1.04 (1.01; 1.08)**	**0.02**	*1*.*05 (1*.*01; 1*.*09)*	*0*.*02*	*1*.*03 (0*.*99; 1*.*08)*	*0*.*15*
Diameter of punctured lesion, per cm	0.96 (0.81; 1.15)	0.67	**0.75 (0.64; 0.87)**	**<0.01**	***0*.*76 (0*.*64; 0*.*90)***	**<0.01**	***0*.*73 (0*.*59; 0*.*90)***	**<0.01**
Pathway from skin to lesion, per cm	1.07 (0.87; 1.32)	0.53	**1.24 (1.05; 1.46)**	**0.01**	***1*.*21 (1*.*01; 1*.*44)***	***0.04***	***1*.*29 (1*.*04; 1*.*60)***	**0.02**
Emphysema	1.07 (0.43; 2.67)	0.88	1.04 (0.52; 2.05)	0.92	*0*.*83 (0*.*39; 1*.*76)*	*0*.*63*	*1*.*58 (0*.*63; 3*.*95)*	*0*.*33*
Repeated biopsies, ref.: 1	1.60 (0.64; 4.04)	0.32	0.61 (0.31; 1.21)	0.16	*0*.*77 (0*.*36; 1*.*65)*	*0*.*51*	***0*.*38 (0*.*15; 0*.*96)***	***0*.*04***

* *binary and*

** *multinomial logistic regression models were calculated*.

**Fig 4:**
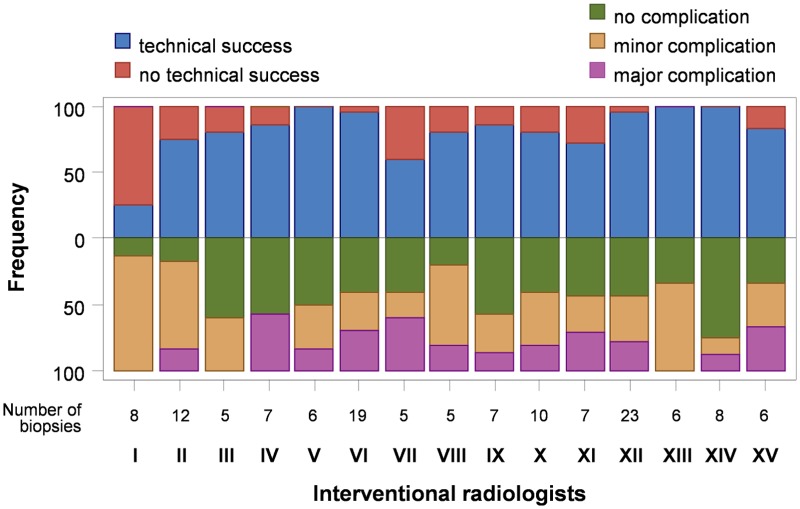
Technical success (upper part) and rate of complications (lower part) by interventional radiologists. There was a significant difference in technical success between interventional radiologists, suggesting that interventional radiologists-related factors strongly affect outcome of percutaneous transthoracic biopsies of pulmonary tumors.

The biopsy procedures in the study population were performed by 15 different interventional radiologists. For both, technical success and rate of complications, we observed differences between the interventional radiologists (subgroup of radiologists with at least 5 biopsies, n = 135 patients; 15 interventional radiologists) (Fig 4). The technical success rate ranged from 25% to 100% and was significantly different between interventional radiologists (p <0.01). The rate of complications ranged from 25% to 88%. However, this was not statistically significant (p = 0.52).

## Discussion

In this study we investigated predictors influencing the technical success and rate of complications of percutaneous transthoracic lung biopsies of potentially malignant pulmonary tumors. Patient-related and intervention-related predictors had no influence on the technical success of percutaneous lung biopsies. However, technical success varied significantly between interventional radiologists. In contrast, the rate of complications was influenced by lesion diameter and length of interventional pathway.

Percutaneous transthoracic lung needle biopsy achieves a high technical success rate of 88–97% [4,8–13]. Our success rate of 84.1% is close to the reported range.

Lesion size has been reported to be a potential predictor of technical success for coaxial fine needle aspiration technique [3,4]. Where comparable with our results, Li et al. compared technical success of coaxial fine needle aspiration for lesions smaller than 1.5 cm (median: 1.20 cm) versus lesions larger than 1.5 cm (median: 3.09 cm) and found a significantly lower technical success rate for smaller lesions (74% versus 97%, p <0.05) [4]. In addition, the site of lesion within the lung has been discussed as another potential predictor influencing technical success of percutaneous transthoracic biopsy [14]. Neither the interventional-related factors nor the patient-related variables influenced technical success in our study results. However, it has to be mentioned that our average lesions were larger than Li’s lesions. Also, Li et al. perfomed fine needle aspirations different to the technique used in our studies. Nevertheless, we believe that a stable technical success rate can be achieved if the indication for biopsies is established by an interdisciplinary tumor board carefully selecting patients. This includes the assessment of the feasibility of a biopsy by an experienced interventional radiologist.

In this study we report on a significant difference of success rates between different interventional radiologists. A possible effect of experience on technical success has been suggested but not supported by data of Montaudon et al. [7]. This may explain the wide range of technical success rates reported in the literature. Our data confirm this assumption as we found significant differences in technical success depending on the interventional radiologist performing the intervention. In our opinion, the skill and experience of the interventional radiologist is a strong predictor.

In agreement to this study, pneumothorax and pulmonary bleeding were the most frequent complications of percutaneous transthoracic lung biopsy [15]. In our study we observed a rate of pneumothoraces of 49.3%. Published rates vary widely, from 8–64% [16–20]. Of these 2–18% of patients required a chest tube [16–20]. This is also in line with our findings. Pulmonary bleeding was less frequent (15%) and hemoptysis was an uncommon minor complication (1.5%). In these cases we did not observed any progression to major complications.

The rate of complications was influenced by several intervention-related factors including lesion diameter, and lesion depth as well as patient-related predictors including age. These results are consistent with previous reports [21–26]. An association of emphysema and a higher rate of pneumothoraces has been controversially discussed [6,27,28]. We have not observed a higher rate of complications in the presence of pulmonary emphysema.

The wide range of reported rates of complications underlines our hypothesis that the rate of complications may also be influenced by interventional radiologist-related factors. We have observed differences in rates of complications between different interventional radiologists. However, they were not statistically significant. More studies are required to prove possible differences in rates of complications between different interventional radiologists.

Unfortunately, our study may have several limitations. First, the study has a retrospective design with a fairly small sample size. Second, in contrast to other publications, percutaneous lung biopsies were performed by a quite high number of interventional radiologists. However, the number of patients per group was too small to show statistically significant differences in rates of complications between different interventional radiologists. Further studies should aim at much higher number s of patients per radiologist. This should be realized in a prospective study design.

In conclusion, the outcome of percutaneous transthoracic biopsies of pulmonary lesions, especially its technical success, is probably influenced by the interventional radiologist performing the procedure. In addition, lesion diameter and length of biopsy pathway were predictors of the rate of complications.
